# Intraocular pressure and its association with ocular biometrics in Iranian children

**DOI:** 10.1016/j.optom.2024.100523

**Published:** 2024-09-21

**Authors:** Hassan Hashemi, Mehdi Khabazkhoob, Samira Heydarian, Mohammad Hassan Emamian, Akbar Fotouhi

**Affiliations:** a Noor Research Center for Ophthalmic Epidemiology, Noor Eye Hospital, Tehran, Iran; b Department of Medical Surgical Nursing, School of Nursing and Midwifery, Shahid Beheshti University of Medical Sciences, Tehran, Iran; c Department of Rehabilitation Science, School of Allied Medical Sciences, Mazandaran University of Medical Sciences, Sari, Iran; d Ophthalmic Epidemiology Research Center, Shahroud University of Medical Sciences, Shahroud, Iran; e Department of Epidemiology and Biostatistics, School of Public Health, Tehran University of Medical Sciences, Tehran, Iran

**Keywords:** Intraocular pressure, Glaucoma, Noncontact tonometer, Population based study

## Abstract

**Purpose:**

To determine the mean value and normative distribution of intraocular pressure (IOP) in children and their association with demographic and ocular biometrics.

**Methods:**

Cluster sampling was done to select the students in urban areas of Shahroud, northeast Iran, while all students living in rural areas were selected. IOP was measured in mmHg using a non-contact tonometer, along with corneal and retinal imaging and ocular biometric measurement.

**Results:**

After applying the exclusion criteria, 9154 eyes of 4580 students were analyzed, of whom 2377 (51.9 %) were boys. The mean age of the participants was 12.35±1.73 years (range: 9–15 years). The mean IOP was 15.58±2.83 (15.47–15.69) in total, 15.31±2.77 (15.17–15.46) in boys, and 15.88±2.86 (15.73–16.03) in girls (*p* < 0.001). The mean IOP was 15.07 and 15.49 in students aged 9 and 15 years, respectively. The mean IOP was 15.7 ± 2.64 (15.58–15.81) in urban and 14.52±4.05 (14.27–14.77) in rural students (*p* < 0.001). In the multiple generalized estimating equation model, IOP had a positive association with female sex (β=0.84, *P* < 0.001), systolic blood pressure (β=0.02, *P* < 0.001), cup volume (β=0.99, *P* < 0.001), corneal thickness (β=0.04, *P* < 0.001) and anterior chamber volume (β=0.007, *P* < 0.001) and a negative association with living in the rural area (β=−0.65, *P* < 0.001), rim area (β=−0.39, *P* < 0.001), and corneal diameter (β=−0.18, *P* = 0.045). Furthermore, individuals with myopia exhibited a significantly higher IOP (β=0.35, *P* < 0.001) compared to those with emmetropia.

**Conclusion:**

This study showed the normative distribution of IOP and its associated factors in children. The results can be used in diagnosis and management of glaucoma.

## Introduction

Intraocular pressure (IOP) has a positive relationship with ocular physiology and pathophysiology.^1^^,^^2^ A high IOP in children not only results in corneal edema and its enlargement as well as changes in the Descemet membrane but it is also a modifiable risk factor for glaucoma.^3^ Glaucoma can cause severe ocular abnormalities, including blindness, in children. It has been reported that 1.2 % of the children in the UK^4^ and 3 % and 7 % of the children in north^5^ and south^6^ India are blind due to glaucoma, respectively. It is obvious that early diagnosis and treatment of this disease in early stages can prevent vision disorders in children.^7^

While glaucoma is now defined based on certain functional and structural changes of the visual system, IOP measurement is one of the most accepted methods for categorizing different types of glaucoma across the world.^8^ Hence, several studies have evaluated the normative distribution of IOP in different populations.^9^, ^10^, ^11^, ^12^, ^13^ The inconsistency in the results of studies, even those conducted in ethnically similar populations,^12^^,^^13^ and the importance of IOP as a modifiable risk factor in the diagnosis and referral of glaucoma highlight the necessity of conducting studies on subjects at different ages from diverse ethnic backgrounds, since these studies can improve our understanding of the prevalence of glaucoma and its risk factors in different parts of the world.

A limited number of studies have evaluated the normative distribution of IOP in children but their results are different considering their demographic and methodological differences.^14^, ^15^, ^16^, ^17^, ^18^ Moreover, no population-based studies have been conducted in Iranian children to date. The aim of the present study was to evaluate the normative distribution of IOP and its association with other biometric variables in children participating in the Shahroud Eye Cohort Study.

## Methods

The present study was part of the second phase of Shahroud Schoolchildren Eye Cohort Study, which was conducted in Shahroud, a city in the northeast of Iran. The methodology of this study has been described in detail elsewhere.^19^ In brief, the first phase of this study was conducted in 2015.

This study entailed the collection of samples from both urban and rural areas in Shahroud. Due to the significant number of students living and studying in the city, a multi-stage cluster sampling method was utilized for the urban student population. In urban areas, each classroom was considered a cluster. In total, 200 clusters were selected from the 473 clusters available in Shahroud proportional to the number of classrooms in each school. In rural areas, the limited student population required the inclusion of all students from the village who were enrolled in local schools, employing a census.

The second phase of this study was conducted in 2018 with a similar setting. All students who partook in phase one were invited to participate in phase two on a predetermined day.

After selecting the students and transferring them to the examination site, they were interviewed to record their demographic data and past medical history. Then, their blood pressure and anthropometric indexes were measured. Optometric examinations included the measurement of visual acuity and refraction. Uncorrected distance visual acuity was measured using the Nidek CP-770 chart projector at 3 m. Then, non-cycloplegic refraction was measured using the Nidek ARK-510A auto refractometer and the results were refined using the Heine Beta 200 retinoscope ((HEINE Optotechnic, Hersching, Germany). Subjective refraction was conducted on students exhibiting unaided visual acuity worse than 20/20.

Finally, all students underwent cycloplegic refraction using cyclopentolate 1 % drops. IOP was measured using a non-contact tonometer (NT-530, NCT Nidek Co., Ltd., Aichi, Japan), before cycloplegia. The IOP measurements were conducted again for 95 students one hour after the initial assessment. Intraclass correlation coefficients (ICC) were then calculated to assess the reliability of the IOP measurements. To calculate ICC, a three-level mixed model was initially fitted. In this model, the IOP measurements were nested within eyes, and eyes were nested within individuals. The ICCs were then defined at the eye level using the “estat icc” command in STATA software (College Station, TX: StataCorp LLC) after running the mixed model.

The Allegro Biograph (WaveLight AG, Erlangen, Germany) was used for biometric measurements and the optical coherence tomography (OCT) (ZEISS Cirrus™ HD-OCT Model 4000 (Carl Zeiss-Meditec, Dublin, CA) was used for macular and optic nerve head imaging. Corneal imaging was done using the Pentacam HR. OCT imaging and IOP measurement were only done in the second phase. OCT imaging for retinal indices was done after cycloplegia with dilated pupils to obtain more accurate images.

## Exclusion criteria

The students with a history of ocular surgery, amblyopia, tropia, a best corrected visual acuity of worse than 20/30 were excluded from the study. The Pentacam images with OK quality and OCT images with SS≥0.6 were included in the study.

## Definitions

Cycloplegic refraction was used to determine refractive error. Similar to previous studies conducted in children,^20^ we also considered a spherical equivalent of equal to or worse than −0.5D as myopia and +2D or worse as hyperopia. A spherical equivalent ranging from −0.49 D to +1.99 D was classified as emmetropia.

### Statistical analysis

The mean, standard deviation (SD), 95 % confidence interval (95 % CI), normal range, and 25 %, 50 %, 75 %, and 95 % percentiles of IOP were reported according to the study variables. The mean value ±2 SD was considered to calculate the normal range. The cluster effect was considered for accurate estimation of standard error, and a sampling weight was applied considering the sampling method in urban and rural areas. Since the results of both eyes were analyzed, simple and multiple generalized estimating equation (GEE) models were used to evaluate the association of IOP with ocular biometrics and other independent variables.

## Results

Of 5620 students who participated in phase one, 5292 partook in phase two. After applying the exclusion criteria, 9154 eyes of 4580 students were analyzed, of whom 2377 (51.9 %) were male. The mean age of the students was 12.35±1.73 years (9–15 years). The ICC recorded were 0.85 (95 % CI: 0.79–0.89), indicating a good reliability in IOP measurements.

Table 1 presents the mean, SD, and 95 % CI of IOP in all subjects according to age and sex. The mean IOP was 15.58±2.83 mmHg (15.47–15.69). Table 2 shows the normal range of IOP in the participants according to age and sex. The normal range of IOP was 9.92 to 21.24 mmHg in all students with a skewness and kurtosis of 0.353 and 0.174, respectively. Table 3 presents the 25 %, 75 %, 95 %, and 99 % percentiles of IOP in the students according to age and sex.

**Table 1:** The mean, standard deviation and 95 % confidence intervals (in parenthesis) of intraocular pressure (mmHg) by age and sex.

Age groups (year)		Total	Male	Female
	Total	15.58 ± 2.83 (15.47–15.69)	15.31 ± 2.77 (15.17–15.46)	15.88 ± 2.86 (15.73–16.03)
	9	15.07 ± 2.71 (14.66–15.48)	14.95 ± 2.79 (14.38–15.51)	15.20 ± 2.60 (14.59–15.81)
	10	15.74 ± 2.68 (15.53–15.94)	15.61 ± 2.65 (15.27–15.95)	15.86 ± 2.70 (15.63–16.10)
	11	15.67 ± 2.73 (15.46–15.87)	15.50 ± 2.87 (15.19–15.81)	15.84 ± 2.57 (15.58–16.10)
	12	15.62 ± 2.81 (15.35–15.89)	15.30 ± 2.72 (14.96–15.64)	15.94 ± 2.86 (15.55–16.32)
	13	15.63 ± 2.82 (15.41–15.85)	15.50 ± 2.74 (15.26–15.74)	15.81 ± 2.91 (15.43–16.20)
	14	15.42 ± 2.93 (15.10–15.74)	14.90 ± 2.74 (14.53–15.27)	16.02 ± 3.02 (15.58–16.46)
	15	15.49 ± 3.00 (15.24–15.74)	15.11 ± 2.79 (14.83–15.38)	15.90 ± 3.16 (15.55–16.24)

**Table 2:** The normal range of Intraocular pressure (mmHg) by age and sex.

Age groups (year)		Total	Male	Female
	Total	9.92 to 21.24	9.77 to 20.85	10.16 to 21.60
	9	9.65 to 20.49	9.37 to 20.53	10.00 to 20.40
	10	10.38 to 21.10	10.31 to 20.91	10.46 to 21.26
	11	10.21 to 21.13	9.76 to 21.24	10.70 to 20.98
	12	10.00 to 21.24	9.86 to 20.74	10.22 to 21.66
	13	9.99 to 21.27	10.02 to 20.98	9.99 to 21.63
	14	9.56 to 21.28	9.42 to 20.38	9.98 to 22.06
	15	9.49 to 21.49	9.53 to 20.69	9.58 to 22.22

**Table 3:** Percentiles of Intraocular pressure (mmHg) by age and sex.

Age and sex groups	Percentiles	25 %	75 %	95 %	99 %
	Total	13.7	17.3	20.3	23.0
Sex	Male	13.3	17.0	20.0	22.7
	Female	14.0	17.3	21.0	23.0
Age	9	13.3	16.7	19.7	21.0
	10	13.7	17.3	20.7	22.7
	11	13.7	17.3	20.3	22.7
	12	13.7	17.3	20.3	22.3
	13	13.7	17.0	20.7	23.3
	14	13.3	17.0	20.7	23.3
	15	13.3	17.0	20.7	23.0

**Fig. 1:**
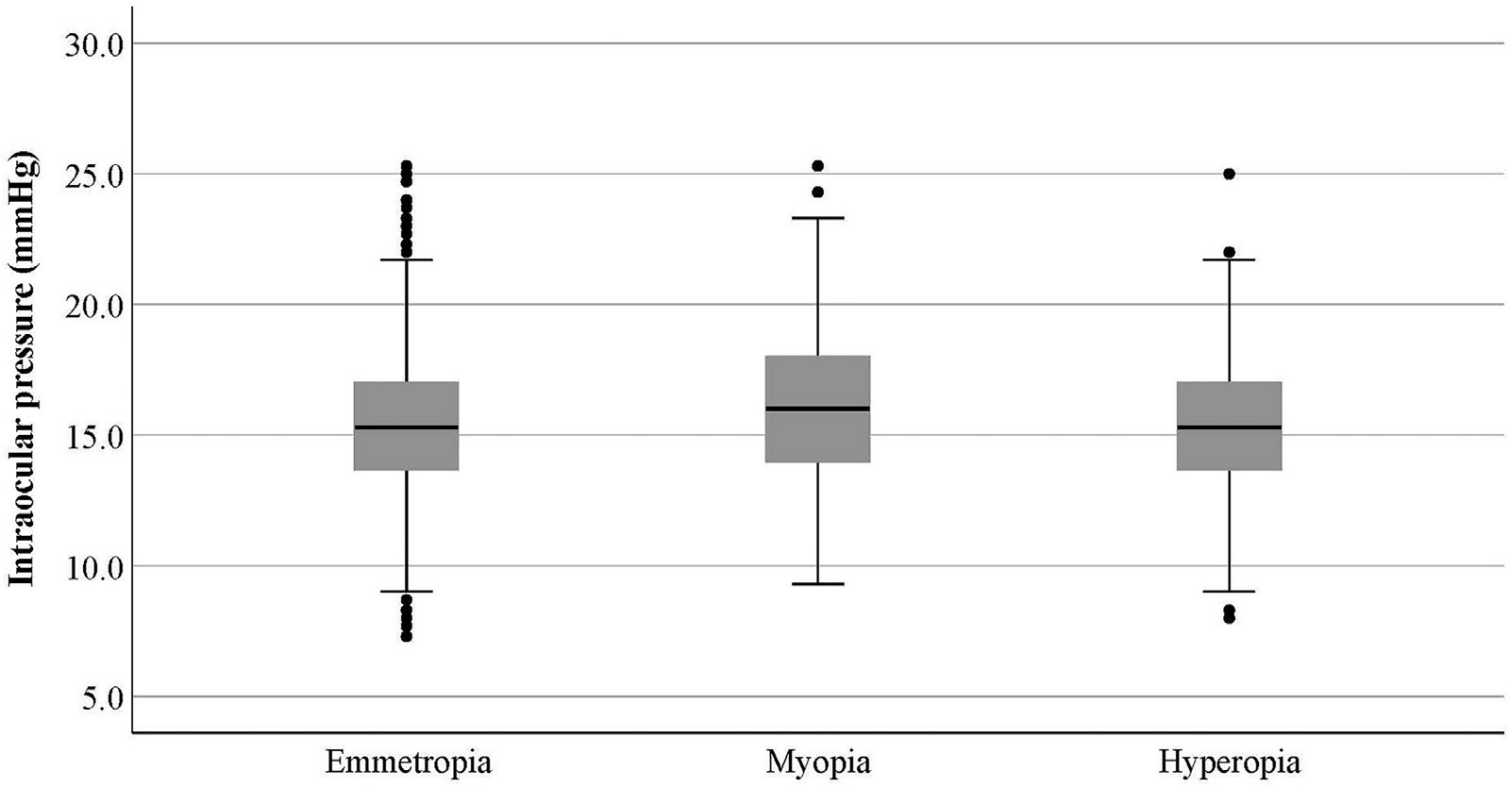
The mean and 95 % confidence interval of intraocular pressure (mmHg) by refractive errors.

According to Table 1, the mean IOP was higher in female students. GEE analysis showed that the difference was significant (*p* < 0.001). IOP changes with age were non-linear. The mean IOP was 15.7 ± 2.64 mmHg (15.58–15.81) in urban and 14.52±4.05 mmHg (14.27–14.77) in rural students. GEE analysis showed that the mean IOP was significantly higher in urban students (*p* < 0.001). Fig. 1 shows the mean IOP according to the refractive error. The lowest and highest IOP was seen in hyperopic and myopic participants, respectively. GEE analysis showed a significantly higher IOP in myopic subjects compared to emmetropic ones (*p* < 0.001) while no difference was observed between myopic and hyperopic individuals (*p* = 0.361).

The association of IOP with age, sex, living place, systolic and diastolic blood pressure, BMI, macular thickness, macular volume, rim area, average vertical cup/disk ratio, disc area, cup volume, central corneal thickness, lens thickness, corneal diameter, anterior chamber depth, anterior chamber volume, anterior chamber angle, mean keratometry reading, and refractive errors was evaluated in a multiple GEE model. Considering the association between variables and to prevent collinearity, a number of variables were not included in the model. The results of the final model are presented in Table 4. The final model's collinearity was assessed, revealing a maximum variance inflation factor (VIF) of 1.69 for the ACV.

**Table 4:** Association of intraocular pressure (mmHg) with demographic and ocular variables in simple and multiple generalized estimating equations models.

		Simple model		Multiple model	
Independent variables		Coefficient (95 %CI)	p-value	Coefficient (95 %CI)	p-value
Sex (Female/male)		0.6 (0.44 to 0.75)	<0.001	0.80 (0.67 to 0.94)	<0.001
Age (year)	9	0		NR	
	10	0.63 (0.11 to 1.15)	0.018		
	11	0.53 (0.01 to 1.05)	0.045		
	12	0.47 (−0.05 to 0.98)	0.077		
	13	0.47 (−0.05 to 0.99)	0.074		
	14	0.29 (−0.23 to 0.81)	0.279		
	15	0.37 (−0.16 to 0.89)	0.169		
Residence Place (Rural/urban)		−1.18 (−1.37 to −0.98)	<0.001	−0.56 (−0.72 to −0.4)	<0.001
Body mass index		0.05 (0.03 to 0.06)	<0.001	NR	
Systolic blood pressure (mm/Hg)		0.01 (0 to 0.02)	0.014	0.01 (0.01 to 0.02)	<0.001
Diastolic blood pressure (mm/Hg)		0.04 (0.03 to 0.05)	<0.001	NR	
Macular thickness (µ)		−0.001 (0 to 0)	0.168	NR	
Macular volume (mm^3^)		−0.06 (−0.14 to 0.02)	0.119	NR	
Rim area (mm^2^)		−0.36 (−0.53 to −0.19)	<0.001	−0.38 (−0.55 to −0.21)	<0.001
Disc area (mm^2^)		0.001 (−0.12 to 0.13)	0.984	NR	
Average vertical cup-disc ratio		0.72 (0.4 to 1.03)	<0.001	NR	
Cup volume (mm^3^)		1.07 (0.66 to 1.49)	<0.001	0.96 (0.57 to 1.36)	<0.001
Central corneal thickness (micron)		0.04 (0.04 to 0.04)	<0.001	0.04 (0.04 to 0.04)	<0.001
Anterior chamber depth (mm)		−0.47 (−0.75 to −0.19)	<0.001	NR	
Lens thickness (mm)		−0.02 (−0.36 to 0.31)	0.888	NR	
Axial length (mm)		0.22 (0.12 to 0.32)	<0.001	NR	
White-to-white corneal diameter (mm)		−0.2 (−0.37 to −0.02)	0.026	−0.19 (−0.37 to 0)	0.045
Anterior chamber volume (mm^3^)		−0.003 (−0.005 to 0)	0.028	0.006 (0.003 to 0.008)	<0.001
Anterior chamber volume (degree)		−0.002 (−0.006 to 0.002)	0.295	NR	
Mean keratometry (diopter)		−0.1 (−0.15 to −0.05)	<0.001	NR	
Refractive errors	Emmetropia	0		0	
	Myopia	0.47 (0.23 to 0.71)	<0.001	0.35 (0.13 to 0.56)	0.001
	Hyperopia	0.15 (−0.17 to 0.47)	0.361	0.11 (−0.19 to 0.4)	0.483

NR: not retained in multiple model.

Multiple model fit: AIC = 36,114.98; BIC = 36,191.94.

The GEE model revealed a positive association between IOP with female sex, systolic and diastolic blood pressures, cup volume, corneal thickness, and anterior chamber volume. In contrast, IOP showed a negative relationship with living in rural regions, rim area, and corneal diameter. Furthermore, individuals with myopia exhibited a significantly higher IOP (β=0.35, *P* < 0.001) compared to those with emmetropia.

**Fig. 2:**
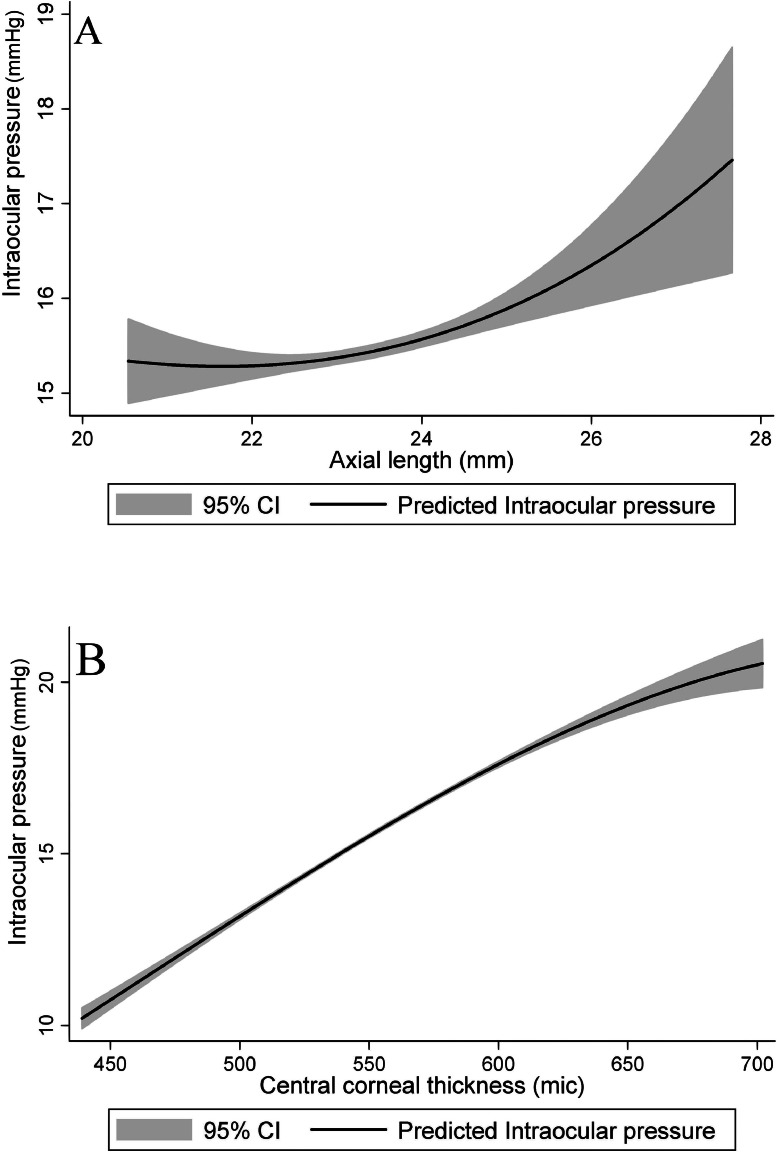
The association of intraocular pressure with axial length (A) and central corneal thickness (B).

Fig. 2 presents the correlation of IOP with central corneal thickness and axial length.

## Discussion

A population-based study was conducted to determine the normative distribution of IOP in a large sample of children aged 9–15 years and its association with a number of biometric variables. A few studies have investigated the distribution of this parameter in children across the world.^15^, ^16^, ^17^, ^18^ The normative distribution of IOP in Iranian children and its association with biometric parameters were investigated in the present study for the first time.

The mean IOP was 15.58±2.83 mmHg in the present study, which was close to the mean IOP of children measured in studies conducted in Iran,^21^ Singapore,^22^ China,^23^^,^^24^ and Malaysia.^25^

However, it was lower than the mean IOP of children measured in studies conducted in Turkey (17.42 and 17.9 mmHg),^26^^,^^27^ East China (17.6 mmHg),^28^ and USA (black children: 19.3 mmHg, White children: 17.7 mmHg).^29^ It should be noted that all of the studies that reported similar IOP values used non-contact methods for IOP measurement while the studies that reported higher values used other methods such as the tono-pen. Moreover, studies that compared tono-pen with non-contact^27^ or rebound tonometry^26^ methods reported a higher mean IOP value with tono-pen compared to other methods. Therefore, in addition to differences in the ethnicity, sample size, and age group across studies, differences in the IOP measurement methods can also affect the distribution of IOP. Additionally, an intriguing factor that may influence IOP measurement is the variability in corneal stiffness among individuals from diverse geographical regions and ethnic backgrounds. This variation could be attributed to genetic differences or the effects of sunlight exposure and natural crosslinking.^30^

In this study, there was no statistically significant relationship between age and IOP based on the multiple GEE model. Although some studies reported different results,^23^^,^^24^^,^^27^ Jiang et al.^28^ found similar findings in children aged 4–18 years. Their research indicated that, although IOP increased until the age of 10 years, a notable decline was recorded between the ages of 10 and 18 years, establishing a positive correlation between elevated IOP and younger age.

The results of the present study showed a significantly higher mean IOP value in female subjects. Although this finding was previously reported in some studies in children^24^^,^^27^^,^^28^ and adults,^12^^,^^31^ some other studies showed no significant inter-gender difference in the mean IOP.^22^^,^^24^^,^^25^ It is difficult to explain the reason for the higher IOP values in girls and more studies are required in this regard.

The mean IOP was higher in urban areas (15.7 ± 2.64 mmHg) versus rural areas (14.52±4.05 mmHg). Studies that have been conducted in this regard have reported different results. While Xu et al. found no correlation between IOP and living place (urban or rural),^32^ Jiang et al. reported similar results to our study.^28^ A positive correlation between IOP and time spent indoors,^28^ which is far more in the urban lifestyle, can be a good reason for the higher IOP value in urban versus rural students.

The analysis conducted using the multiple GEE model did not reveal a significant association between axial length and IOP. The literature is contradictory in this regard.^33^ While some studies found a significant positive correlation between axial length and IOP in children,^33^^,^^34^ some other failed to find such a relationship.^26^^,^^35^ Saeedi et al. found a positive relationship between IOP and axial length in an adult population.^36^ In their study, AL was measured in 21 patients before and after IOP reduction following trabeculectomy. The results showed a decrease in AL after IOP reduction, which could be due to reduced mechanical pressure on ocular tissues like the sclera and cornea resulting in scleral relaxation and axial length reduction. Therefore, the relationship between higher IOP values and longer ALs in this study could be due to increased biomechanical pressure on the globe wall resulting in ocular enlargement in subjects with higher IOP values.

Several studies evaluated the relationship between IOP and refractive errors in children^28^^,^^37^ and many of them found a positive correlation between IOP and myopia development and progression.^28^^,^^37^

The findings of this study indicated that the average IOP in individuals with myopia was significantly elevated in the final model, which is consistent with previous research.

In a study by Jensen et al.,^38^ children aged 9–12 years that had higher baseline IOP values, had higher rates of myopia progression, indicating a positive association between IOP and myopia development. IOP reduction can slow down scleral remodeling and myopia progression through reducing mechanical pressure on the sclera and choroidal vessels resulting in increased choroidal blood flow and reduced scleral hypoxia.^39^

In line with the present study, a comparable investigation involving Iranian children aged 6 to 12 years reported that IOP was elevated in those with myopia.^21^

CCT was another parameter that was evaluated in the present study. A significant positive correlation was found between IOP and CCT, which is consistent with the results of several studies in children and adults.^25^^,^^26^^,^^29^ Tong et al.^40^ and Li et al.^24^ conducted studies in children with similar age ranges and measurement methods to the present study and found a positive correlation between IOP and CCT. This positive relationship can result from the effect of the corneal tissue stiffness or softness on IOP estimation by the tonometer; in other words, the thicker or stiffer the cornea, the higher the measured IOP. Kass et al. found that tonometry underestimates IOP by up to 5 mmHg in thin corneas and overestimates IOP by as much as 7 mmHg in thick corneas.^41^ Therefore, more studies are required to evaluate the effect of CCT in estimating true IOP.

Hypertension is a known risk factor for high IOP.^42^^,^^43^ Several studies have shown a positive relationship between IOP and systolic blood pressure^42^^,^^44^^,^^45^ while fewer studies have reported a relationship between IOP and diastolic blood pressure.^42^^,^^43^ The present study found a significant positive correlation between IOP and systolic blood pressure. Although the exact mechanism of increased IOP in hypertension is not clearly understood, increased production of the aqueous humour with ultrafiltration due to raised ciliary artery pressure or increased stimulation of the sympathetic system with a rise in the serum corticosteroid level, which can cause a simultaneous elevation in the blood pressure and IOP, may be the reason for this finding.^46^ Considering the fact that blood pressure is a modifiable factor and high IOP is one of the most important risk factors of glaucoma, blood pressure can be potentially considered a modifiable risk factor for glaucoma.

Several studies have evaluated the effect of elevated IOP on optic nerve head changes like the cup to disc ratio in animals^47^ and humans.^48^ In 1998, Azuara et al. found that short-term increase of IOP in a healthy eye raised eye wall mechanical stress, caused displacement of the optic nerve head tissues, and increased the cup volume.^48^ IOP is an important determinant of the cup volume changes. The Blue Mountain study is a well-known study in this regard which showed that each 10-mmHg elevation in the IOP increased the cup to disc ratio by as much as 0.04.^49^ Moreover, Klein et al. evaluated optic disc changes during five years. The results showed a significant positive correlation between IOP and cup volume.^50^ Although the above studies were conducted in adult populations, the present study also revealed a significant positive correlation between IOP and cup volume in children.

Several studies have shown that the neuroretinal rim area is significantly less in patients with elevated IOP compared to healthy subjects.^51^ The present study found a significant negative relationship between IOP and rim area. Pardon et al. conducted a study on macaques and reported similar results. They found that short-term increase of IOP by 25 and 40 mmHg for two hours reduced the neuroretinal rim area. They also reported that rim area changes were relatively reversible by reducing the IOP to 10 mmHg in these primates.^52^ In another study by Siaudvytyte et al., although the neuroretinal rim area was smaller in patients with high-pressure glaucoma compared to healthy subjects, which confirms our results, patients with normal tension glaucoma had the smallest neuroretinal rim area compared to the other two groups indicating the effect of a unknown determinant other than IOP requiring further research.^53^

Considering the importance of IOP in detection of glaucoma and given the fact that the normative distribution of IOP is not similar across geographical regions and in different age groups, the results of the present study can provide an appropriate criterion for detection of this disease in children. In the present study, non-contact tonometry was used for IOP measurement. Hence, considering the marked difference in IOP between non-contact tonometry and other methods, IOP measurement with Goldmann applanation tonometry (GAT) may produce different results. However, it was not possible to use GAT in the present study due to the limitations associated with its use in children.

## Declaration of competing interest

The authors declare that they have no conflicts of interest.

## References

[1] Stamper R.L. (2011). A history of intraocular pressure and its measurement. Optom Vis Sci.

[2] Sanchez I., Martin R. (2019). Advances in diagnostic applications for monitoring intraocular pressure in Glaucoma: a review. J Optom.

[3] Marchini G., Toscani M., Chemello F. (2014). Pediatric glaucoma: current perspectives. Pediatric Health, Med Therapeut.

[4] Durnian J.M., Cheeseman R., Kumar A. (2010). Childhood sight impairment: a 10-year picture. Eye (Lond).

[5] Bhattacharjee H., Das K., Borah R.R. (2008). Causes of childhood blindness in the northeastern states of India. Indian J Ophthalmol.

[6] Dorairaj S.K., Bandrakalli P., Shetty C. (2008). Childhood blindness in a rural population of southern India: prevalence and etiology. Ophthalmic Epidemiol.

[7] Solebo A.L., Rahi J. (2014). Epidemiology, aetiology and management of visual impairment in children. Arch Dis Child.

[8] McManus J.R., Netland P.A. (2013). Screening for glaucoma: rationale and strategies. Curr Opin Ophthalmol.

[9] Dielemans I., Vingerling J.R., Wolfs R.C. (1994). The prevalence of primary open-angle glaucoma in a population-based study in The Netherlands. The Rotterdam Study. Ophthalmology.

[10] Giuffre G., Giammanco R., Dardanoni G., Ponte F. (1995). Prevalence of glaucoma and distribution of intraocular pressure in a population. The Casteldaccia eye study. Acta Ophthalmol Scand.

[11] Klein B.E., Klein R., Sponsel W.E. (1992). Prevalence of glaucoma. The Beaver dam eye study. Ophthalmology.

[12] Hashemi H., Kashi A.H., Fotouhi A., Mohammad K. (2005). Distribution of intraocular pressure in healthy Iranian individuals: the Tehran Eye Study. Br J Ophthalmol.

[13] Lee J.S., Lee S.H., Oum B.S. (2002). Relationship between intraocular pressure and systemic health parameters in a Korean population. Clin. Experiment. Ophthalmol.

[14] Goss D.A., Caffey T.W. (1999). Clinical findings before the onset of myopia in youth: 5. Intraocular pressure. Optometry Vision Sci: Official Publication Am Acad Optometry.

[15] Han F., Li J., Zhao X. (2021). Distribution and analysis of intraocular pressure and its possible association with glaucoma in children. Int Ophthalmol.

[16] Sakalar Y.B., Keklikci U., Unlu K. (2012). Distribution of central corneal thickness and intraocular pressure in a large population of Turkish school children. Ophthalmic Epidemiol.

[17] Zhou Q., Gao T.Y., Fan S.J. (2022). Intraocular pressure, age, and central corneal thickness in a healthy Chinese children population: the handan offspring myopia study. Ophthalmic Epidemiol.

[18] Pileggi C., Papadopoli R., De Sarro C. (2021). Obesity, blood pressure, and intraocular pressure: a cross-sectional study in Italian children. Obes Facts.

[19] Emamian M.H., Hashemi H., Khabazkhoob M. (2019). Cohort profile: shahroud schoolchildren eye cohort study (SSCECS). Int J Epidemiol.

[20] Hashemi H., Khabazkhoob M., Fayaz M. (2023). Refractive errors and their associated factors in schoolchildren: a structural equation modeling. Ophthalmic Epidemiol.

[21] Masoumpour M.B., Nowroozzadeh M.H., Talebnejad M.R. (2020). Distribution of intraocular pressure in healthy Iranian children: the Shiraz pediatric eye study. J aapos.

[22] Lim L., Cheung N., Gazzard G. (2009). Corneal biomechanical properties and retinal vascular caliber in children. Invest Ophthalmol Vis Sci.

[23] Lee A.J., Saw S.M., Gazzard G. (2004). Intraocular pressure associations with refractive error and axial length in children. Br J Ophthalmol.

[24] Li S., Li S.M., Wang X.L. (2017). Distribution and associations of intraocular pressure in 7- and 12-year-old Chinese children: the Anyang childhood eye study. PLoS ONE.

[25] Heidary F., Gharebaghi R., Wan Hitam W.H. (2011). Central corneal thickness and intraocular pressure in Malay children. PLoS ONE.

[26] Sahin A., Basmak H., Yildirim N. (2008). The influence of central corneal thickness and corneal curvature on intraocular pressure measured by tono-pen and rebound tonometer in children. J Glaucoma.

[27] Yildirim N., Sahin A., Basmak H., Bal C. (2007). Effect of central corneal thickness and radius of the corneal curvature on intraocular pressure measured with the Tono-Pen and noncontact tonometer in healthy schoolchildren. J Pediatr Ophthalmol Strabismus.

[28] Jiang W.J., Wu J.F., Hu Y.Y. (2014). Intraocular pressure and associated factors in children: the Shandong children eye study. Invest Ophthalmol Vis Sci.

[29] Muir K.W., Duncan L., Enyedi L.B., Freedman S.F. (2006). Central corneal thickness in children: racial differences (black vs. white) and correlation with measured intraocular pressure. J Glaucoma.

[30] Komninou M.A., Seiler T.G., Enzmann V. (2024). Corneal biomechanics and diagnostics: a review. Int Ophthalmol.

[31] Hashemi H., Khabazkhoob M., Emamian M.H. (2016). Distribution of intraocular pressure and its determinants in an Iranian adult population. Int J Ophthalmol.

[32] Xu L., Li J., Zheng Y. (2005). Intraocular pressure in Northern China in an urban and rural population: the Beijing eye study. Am J Ophthalmol.

[33] Lee S.M., Edwards M.H. (2000). Intraocular pressure in anisometropic children. Optom Vis Sci.

[34] Chua J., Tham Y.C., Liao J. (2014). Ethnic differences of intraocular pressure and central corneal thickness: the Singapore epidemiology of eye diseases study. Ophthalmology.

[35] Memarzadeh F., Ying-Lai M., Azen S.P. (2008). Associations with intraocular pressure in Latinos: the Los Angeles Latino eye study. Am J Ophthalmol.

[36] Saeedi O., Pillar A., Jefferys J. (2014). Change in choroidal thickness and axial length with change in intraocular pressure after trabeculectomy. Br J Ophthalmol.

[37] Quinn G.E., Berlin J.A., Young T.L. (1995). Association of intraocular pressure and myopia in children. Ophthalmology.

[38] Jensen H. (1992). Myopia progression in young school children and intraocular pressure. Doc Ophthalmol.

[39] Wang P., Chen S., Liu Y. (2021). Lowering intraocular pressure: a potential approach for controlling high myopia progression. Invest Ophthalmol Vis Sci.

[40] Tong L., Saw S.M., Siak J.K. (2004). Corneal thickness determination and correlates in Singaporean schoolchildren. Invest Ophthalmol Vis Sci.

[41] Kass M.A. (1996). Standardizing the measurement of intraocular pressure for clinical research. Guidelines from the Eye Care technology Forum. Ophthalmology.

[42] Yasukawa T., Hanyuda A., Yamagishi K. (2022). Relationship between blood pressure and intraocular pressure in the JPHC-NEXT eye study. Sci Rep.

[43] Plotnikov D., Huang Y., Khawaja A.P. (2022). High blood pressure and intraocular pressure: a mendelian randomization study. Invest Ophthalmol Vis Sci.

[44] Bonomi L., Marchini G., Marraffa M. (2000). Vascular risk factors for primary open angle glaucoma: the Egna-Neumarkt Study. Ophthalmology.

[45] Foster P.J., Machin D., Wong T.Y. (2003). Determinants of intraocular pressure and its association with glaucomatous optic neuropathy in Chinese Singaporeans: the Tanjong Pagar Study. Invest Ophthalmol Vis Sci.

[46] Carel R.S., Korczyn A.D., Rock M., Goya I. (1984). Association between ocular pressure and certain health parameters. Ophthalmology.

[47] Chauhan B.C., Pan J., Archibald M.L. (2002). Effect of intraocular pressure on optic disc topography, electroretinography, and axonal loss in a chronic pressure-induced rat model of optic nerve damage. Invest Ophthalmol Vis Sci.

[48] Azuara-Blanco A., Harris A., Cantor L.B. (1998). Effects of short term increase of intraocular pressure on optic disc cupping. Br J Ophthalmol.

[49] Healey P.R., Mitchell P., Smith W., Wang J.J. (1997). The influence of age and intraocular pressure on the optic cup in a normal population. J Glaucoma.

[50] Klein B.E., Klein R., Jensen S.C. (1997). Changes in the optic disc over a five-year interval: the Beaver Dam Eye Study. Curr Eye Res.

[51] Airaksinen P.J., Tuulonen A., Alanko H.I. (1992). Rate and pattern of neuroretinal rim area decrease in ocular hypertension and glaucoma. Arch Ophthalmol.

[52] Pardon L.P., Harwerth R.S., Patel N.B. (2020). Neuroretinal rim response to transient changes in intraocular pressure in healthy non-human primate eyes. Exp Eye Res.

[53] Siaudvytyte L., Januleviciene I., Ragauskas A. (2014). The difference in translaminar pressure gradient and neuroretinal rim area in glaucoma and healthy subjects. J Ophthalmol.

